# A palaeoclimate proxy database for water security planning in Queensland Australia

**DOI:** 10.1038/s41597-021-01074-8

**Published:** 2021-11-02

**Authors:** Jacky Croke, John Vítkovský, Kate Hughes, Micheline Campbell, Sahar Amirnezhad-Mozhdehi, Andrew Parnell, Niamh Cahill, Ramona Dalla Pozza

**Affiliations:** 1 School of Geography, University College Dublin, Belfield, Dublin, Ireland; 2 Queensland Department of Environment and Science, Brisbane, Australia; 3 Catchment Connections, Brisbane, Australia; 4 Hamilton Institute, Mathematics and Statistics, Maynooth University, Maynooth, Ireland; 5 Victoria Department of Environment, Land, Water and Planning, Melbourne, Australia

**Keywords:** Palaeoclimate, Hydrology

## Abstract

Palaeoclimate data relating to hydroclimate variability over the past millennia have a vital contribution to make to the water sector globally. The water industry faces considerable challenges accessing climate data sets that extend beyond that of historical gauging stations. Without this, variability around the extremes of floods and droughts is unknown and stress-testing infrastructure design and water demands is challenging. User-friendly access to relevant palaeoclimate data is now essential, and importantly, an efficient process to determine which proxies are most relevant to a planning scenario, and geographic area of interest. This paper presents PalaeoWISE (**Palaeo**climate Data for **W**ater **I**ndustry and **Se**curity Planning) a fully integrated, and quality-assured database of proxy data extracted from data repositories and publications collated in Linked Paleo Data (LiPD) format. We demonstrate the application of the database in Queensland, one of Australia’s most hydrologically extreme states. The database and resultant hydroclimate correlations provides both the scientific community, and water resource managers, with a valuable resource to better manage for future climate changes.

## Abstract (summary)

**Table:** 

Measurement(s)	climate
Technology Type(s)	digital curation
Factor Type(s)	proxy type • geographic location • temporal interval • environmental material
Sample Characteristic - Environment	climate system
Sample Characteristic - Location	Earth (planet)

Machine-accessible metadata file describing the reported data: 10.6084/m9.figshare.16607162

## Background & Summary

The essential value of high-resolution accessible global palaeoclimate datasets to climate change predictions is well recognised^1–3^. The rise in popularity of data repositories together with advances in computing mean that large-scale data compilation and analyses are now more accessible^1,2,4–7^. Despite such advances, a disconnect remains between the availability of palaeoclimate databases and uptake by key industry sectors. One such sector is the water industry, which faces significant challenges with respect to climate variability and change and its impact on future water supply^8^.

Improvements to industry decision-making can only be facilitated by establishing the ‘plausible ranges of climate change’^8^ and the reduction in the uncertainty afforded by millennial-scale records^9^. The relatively short observational record-length (<100 years) available for hydrological modelling and water planning, is insufficient to capture variability around the extremes of floods and droughts^9–14^. Climate information also plays a key role in enabling the sort of ‘smarter solutions’ required of the industry, with several applications demonstrating the tangible benefits of incorporating palaeoclimate data into water management^13,15–17^. Palaeoflood data, for example, is now routinely used to improve flood frequency analysis in several countries^9,18,19^ and is especially valuable to ‘stress test’ infrastructure design to safeguard against dam overspill.

Using palaeoclimate data from the Australasian region, we present an efficient and integrated tool that allows access to a standardised database to rapidly assess the proxy records most relevant to a hydroclimate scenario, and geographic area of interest. The database represents an expansion on previous compilations and includes records reported in Freund *et al*. (2017), Dixon *et al*., (2017), and Comas-Bru *et al*., (2020) with additional records sourced directly from publications or authors. The database comprises 396 records derived from 11 different archive types (e.g., corals, tree rings, sediments, speleothems) with an emphasis on the Common Era (i.e., the last 2000 years). We demonstrate the application of this palaeoclimate information to both the scientific community and the water industry by testing the temporal correlation between sample proxy records and a full suite of hydroclimate indices relevant to water planning in Queensland, one of Australia’s largest and climatically variable states. The approach provides palaeoclimatologists, hydrological modellers, water managers, and decision makers with the opportunity to incorporate ranges of environmental change and hydroclimate variability to better inform stress testing decisions. The approach can be used to produce similar output for the entire continent of Australia and elsewhere in the southern hemisphere. The resultant datasets also offer the scientific community a valuable opportunity to explore underlying patterns in the mechanisms driving climate variability in the southern hemisphere.

## Methods

All data presented in this database have previously been published, and the original peer-reviewed publications should be consulted for detailed information on data collection methods, analyses and interpretation. In particular, we stress the importance of recognising some of the inherent limitations of different palaeoclimate proxy data as they relate specifically to chronological uncertainties, and any lagged response between proxy and climate that may be related to site-specific environmental conditions^20^. Some of these limitations are summarised in more detail on the project website www.palaeoclimate.com.au.

### Palaeoclimate data compilation

#### Data Sources

The majority of proxy records were sourced from online data repositories (e.g. NOAA World Data Service for Paleoclimatology, PANGAEA) and extracted using record details contained within the published reviews of Freund *et al*. (2017) and Dixon *et al*. (2017), which focus on proxies relevant to Australian climate. Freund *et al*. (2017) report details of a high-resolution (annual or higher) proxy network from the southern hemisphere which were used to reconstruct rainfall for Australia’s eight natural resource management regions. Low-resolution proxies (>annual) were largely sourced from Dixon *et al*. (2017), who identified a total of 132 high quality palaeoclimate datasets and also provided alternative chronologies based on revised age modelling. Relevant records from the Speleothem Isotopes Synthesis and AnaLysis (SISAL) database^21^ were filtered using the geographic extent for the region influential to Australasian climate (cf. Dixon *et al*. 2017). Where data were not in an online repository, they were sourced from the supplementary materials or directly from the authors.

#### Selection Criteria

Extracted records were screened against several broad criteria to capture the maximum number of both high and low-resolution records before being collated in the database. To enhance usage by water resource managers, the Common Era was prioritised where resolution is generally high, with >50% of datasets having a temporal resolution of annual or greater.

The following final criteria were used:

1. The proxy record must be detailed in a peer-reviewed publication.
2. The proxy record must contain at least two samples dated to within the last 2000 years.
3. The proxy record must span at least 20 years.
4. The proxy record must not require further processing to yield a chronological time series. This relates particularly to the exclusion of tree-ring datasets comprised of raw tree-ring width values, which would require further processing.
5. The proxy must be related directly, or teleconnected to, Australian climate, as stated in the original publication or a more recent published synthesis.

#### Database collation of proxy records

Proxy records including all associated metadata were compiled and reformatted in the Linked Paleo Data (LiPD) format^7^ using the *lipdR* and *dplyr* packages in the statistical language *R*^22–24^. The LiPD format is based on linked JavaScript Object Notation (JSON-ld), and has the benefits of being highly flexible, self-contained (data and metadata are always stored together), and permits integration and comparison with previously published syntheses^1,2,4,25^.

Table 1 outlines a subset of metadata fields for proxy records stored in the database, which is provided as both LiPD and R data files^26^. PalaeoWISE database users are directed to McKay and Emile-Geay (2016) and the Linked Earth Ontology^27^ for full details of database structure and standard definitions and terminology of field names. All included fields are fully described in the PalaeoWISE files^26^. PalaeoWISE^26^ also includes an overview of the completeness of the database fields in the supplementary material (Section 1). Meta-analysis and visualisation of the database were undertaken in *R* using the packages *dplyr*, *ggplot2*, *sf*, and *rnaturalearth*^23,24,28–31^.

**Table 1:** Description of a selection of metadata fields with examples given for the eleven proxy datasets used in the technical validation section.

Brief Citation	DOI	Dataset ID	Location	Latitude	Longitude	Archive Type	Proxy Type	Start Year (CE)	End Year (CE)	Overlap with 1 ka (years)	Continuity	Resolution
Duncan *et al*., 2010^138^	10.1007/s00382-010-0794-2	156	South Island and Lower North Island	−43.27	172.18	Tree Ring	Tree Ring Width	1457	1999	542.0	Continuous	Annual
Barr *et al*., 2019^139^	10.1038/s41598-019-38626-3	199	Swallow Lagoon	−27.50	153.45	Leaf Material	Delta Leaf (Carbon Isotope Discrimination)	−5743	1993	974.0	Continuous With Gaps	Multi-Annual
Hendy *et al*., 2003^140^	10.1191/0959683603hl606rp	269	Great Barrier Reef	−18.50	146.75	Coral	Luminescence	1612	1985	373.0	Continuous	Annual
Griffiths *et al*., 2016141	10.1038/ncomms11719	408	Liang Luar Cave	−8.53	120.43	Speleothem	Delta-Carbon-13	−20	1997	978.0	Continuous	Multi-Annual
Dixon *et al*., 20174	10.5194/cp-13-1403-2017	470	Lake Logung, East Java	−8.04	113.31	Sediment	Calcium-Titanium Ratio	1975	2007	32.5	Continuous	Sub-Annual
Dixon *et al*., 20174	10.5194/cp-13-1403-2017	497	Makassar Strait	3.88	119.45	Foraminifera	Aluminium-Calcium Ratio	1664	1971	306.8	Continuous	Multi-Annual
Jones *et al*., 2014142	10.5194/cp-10-1253-2014	595	Siple Dome	−81.66	−148.72	Ice Core	Delta-Oxygen-18	1919	1995	76.0	Continuous With Gaps	Seasonal

The extended version of this table is included in PalaeoWISE^26^ which details all records in the database.

Following collation and standardisation of proxy records, summary dashboards were produced for each record to facilitate the quality control of database contents similar to those outlined by PAGES2k Consortium (2017). Further detail on quality control procedures and examples of dashboards are provided in the Technical Validation section.

## Data Records

**Fig. 1:**
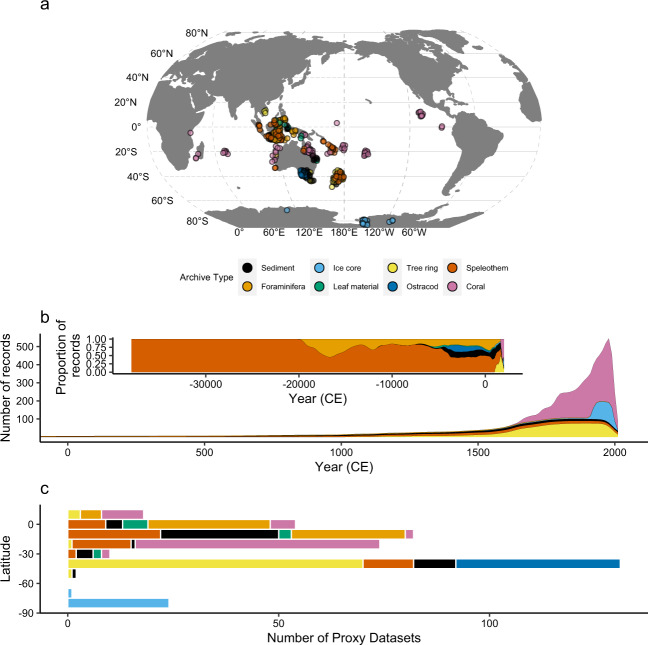
Spatiotemporal overview of the palaeoclimate proxy database (n = 396). (**a**) Distribution of proxy records by archive type. (**b**) Proxy temporal availability by archive type for the Common Era, and proportional availability by archive type for the last~38 ka (inset). (**c**) Latitudinal distribution of proxies by archive type (10 degree bins). Vector map data sourced from http://www.naturalearthdata.com/. An interactive map of the database is available at www.palaeoclimate.com.au.

The PalaeoWISE (**Palaeo**climate Data for **W**ater **I**ndustry and **Se**curity Planning) database contains 396 palaeoclimate proxy records^26,32–128^, each of which documents an archive’s response to past changes in climate. The majority of proxies come from sites located in the Australasian region, with some records in the Indian and central Pacific Oceans, as well as Antarctica (Fig. 1). The geographic distribution of proxies is predominantly from tropical latitudes (Fig. 1). This reflects both the dominance of tropical coral as a palaeoclimate archive for the Australasian region and the influence of dedicated ocean/atmospheric climate research programs that have produced multiple proxy records from a single site (e.g. Global Tropical Moored Buoy Array Program) (Table 2). A single marine sediment core extracted from the Makassar Strait, Indonesia, for example, has yielded four proxy datasets^94^. Records are derived from diverse archives (coral, foraminifera, ice cores, leaf material, ostracods, sediment, speleothems, and tree rings) and the temporal resolutions range from monthly/seasonal (e.g. corals) to decadal/centennial (e.g. foraminifera) (Fig. 1). Records in the database have timespans ranging from 21 to 40,000 years, although the majority of records do not extend beyond the beginning of the Common Era (Fig. 1, Table 2).

**Table 2:** Summary of all proxy records in the database by archive type.

Archive type	References*	No. of datasets	Resolution	Overlap with the Common Era (years)
Coral	Lough, 2011^143^, Tudhope, 2001^144^, Linsley *et al*., 2006^145^, Linsley *et al*., 2000^146^, Urban *et al*., 2000^147^, Zinke *et al*., 2004^148^, Zinke *et al*., 2016^149^, Kuhnert *et al*., 2000^150^, Dunbar *et al*., 1994^151^, Bagnato *et al*., 2005^152^, Linsley, 2000^153^, **Hendy** ***et al****.***, 2003**^140^, Quinn *et al*., 1998^154^, Zinke *et al*., 2015^155^, Charles *et al*., 2003^156^, Cole *et al*., 2000^157^, Kuhnert *et al*., 1999^158^	78	Annual, Monthly/seasonal	402
Foraminifera	Newton *et al*. 2006^159^, Stott *et al*. 2004^160^, Oppo *et al*. 2009^161^, Steinke *et al*. 2014^162^, **Dixon** ***et al****.* **2017**^4^	61	Annual, Decadal/centennial	1987
Ice core	Vance *et al*., 2013^163^, ***Jones et al., 2014***^142^, Banta *et al*., 2008^164^	25	Annual, Monthly/seasonal	1009
Leaf material	***Barr et al., 2019***^139^, Konecky *et al*. 2013^165^, Tierney *et al*. 2010^166^, Langton *et al*. 2008^167^, Dixon *et al*. 2017^4^	11	Annual, Decadal/centennial	2000
Ostracod	Gouramanis *et al*. 2010^168^, Dixon *et al*. 2017^4^	39	Decadal/centennial	2000
Sediment	Marx *et al*., 2011^169^, *Lam et al., 2017*^9^, *Croke et al., 2016*^12^, *Brooke et al., 2008*^85^, Rodysill *et al*. 2012^170^, Saunders *et al*. 2013^171^, Saunders *et al*. 2012^172^, Wilkins *et al*. 2013^173^, Steinke *et al*. 2014^174^, Langton *et al*. 2008^167^, Kemp *et al*. 2012^175^, **Dixon** ***et al****.* **2017**^4^	48	Annual, Decadal/centennial	2011
Speleothem	*Haig et al., 2014*^176^, Rasbury and Aharon, 2006^177^, **Griffiths** ***et al****.* **2016**^141^, Dixon *et al*. 2017^4^, Partin, 2013^178^, Maupin, 2014^179^, Hartmann, 2013^180^, Treble, 2005^181^, Wurtzel, 2018^182^, Chen, 2016^183^, Krause, 2019^184^, Williams, 2005^185^, Williams, 2004^186^, Lorrey, 2008^187^, Griffiths, 2009^188^, Ayliffe, 2013^189^, Nott, 2007^190^, Partin, 2007^191^	59	Annual, Decadal/centennial, Monthly/seasonal	2011
Tree ring	**Duncan** ***et al****.***, 2010**^138^, D’Arrigo *et al*., 1996^192^, Xiong and Palmer, 2000^193^, Palmer *et al*., 1988^194^, Palmer *et al*., 2015^195^, Ahmed and Ogden, 1985^196^, Fowler *et al*., 2004^197^, Fowler, 2008^198^, Buckley *et al*., 1997^199^, Allen *et al*., 2001^200^, O’Donnell *et al*., 2015^201^, Buckley *et al*., 2010^202^, Brookhouse *et al*., 2008^203^, D’Arrigo *et al*., 1998^204^, D’Arrigo *et al*., 2000^205^, Xiong et al., 1998^206^, Norton 1983^207^	75	Annual	981

Note: a single reference may be associated with multiple datasets.

*bold text denotes references for the example datasets discussed in this paper. Italicised text denotes references for which data were sourced from supplementary materials or directly from authors.

**Fig. 2:**
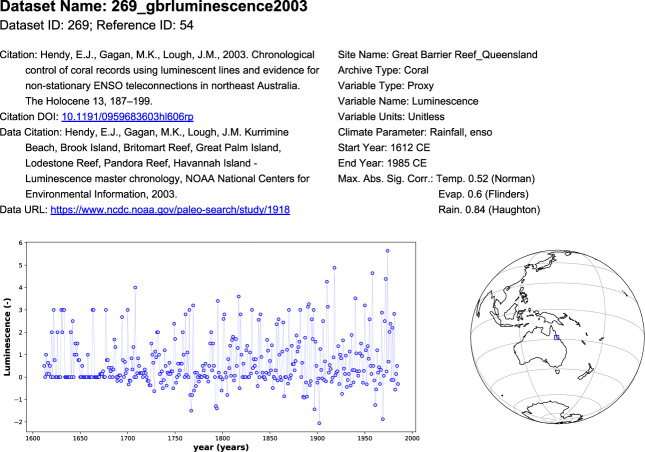
Quality control dashboard for Dataset ID 269. Dashboards for all proxy records in the database are provided in PalaeoWISE^26^.

PalaeoWISE^26^ is hosted on figshare (10.6084/m9.figshare.14593863.v3), which is also accessible via the project website (www.palaeoclimate.com.au/project-outputs/proxy-map/access-the-palaeowise-database/). PalaeoWISE^26^ includes 15 items as detailed in Table 3, together with the code to produce the figures presented in this manuscript. The proxy data are presented as a zipped folder of LiPD and Rdata files and includes a brief introduction on how to interact with LiPD files in R and a README.txt file. PalaeoWISE^26^ also includes all proxy dashboard figures (Fig. 2), and correlation maps and coefficients for each of the 396 proxy records, 73 Queensland catchments, and 75 climate variables. An analysis of correlation coefficient lags (in years) for the seven example proxy datasets is also included in PalaeoWISE^26^. More information for each item can be found in Table 3 and in the PalaeoWISE readme file^26^. The proxy data contained in PalaeoWISE^26^ is also hosted by NOAA World Data Service (WDS) for Paleoclimatology (https://www.ncdc.noaa.gov/paleo/study/34073)^32^. This community-specific, open access repository archives the PalaeoWISE proxy data in LiPD format, and also in the WDS template text format for records not previously archived in the WDS Paleoclimatology^32^.

**Table 3:** Description of files contained in PalaeoWISE^26^.

Filename	Contents
Dataset_details.pdf	Summary table of key metadata for each dataset
lipds.zip	LiPD files of data and metadata for each dataset.
lipds.rdata	Rdata file of data and metadata for each dataset
fieldnames.xlsx	Spreadsheet of fieldnames and their descriptions.
corr_maps.zip	Correlation maps of maximum significant absolute correlation coefficient by catchment for each climate variable and the 396 proxy datasets in the database.
Success_histograms.pdf	PDF of ‘success histograms’ for each climate variable.
Corrs_max_abs_sig.zip	Concise correlations (maximum significant absolute correlation coefficient) for each catchment, dataset, and climate variable.
Corrs_all_lags_sig.zip	Full Correlation data detailed for all lags (−5 to +5) for each catchment, dataset, and climate variable.
For_gis_sig.zip	Concise correlation data formatted for making correlation maps
Data_dashboards.pdf	Dashboards for all proxy datasets
Supplementary_Material.pdf	Results from correlation method comparison.
Croke2021Figs	R code and data to reproduce the figures in this paper
Lipd_guide.html	.html with instructions and examples about reading LiPD files and do some basic manipulation
Lipd_guide.Rmd	Markdown file with instructions and examples about reading and manipulating LiPD files. The code interacts with the data in PalaeoWISE, so users can use the code directly.
README.txt	A text file which details the contents of PalaeoWISE and the structure of the LiPD files

## Technical Validation

### Database quality control

Essential quality assurance was completed on the individual proxy records using summary dashboards following the example of PAGES2k Consortium (2017). Proxy records, which comprise a single timeseries and multiple metadata fields, were verified by comparison with the original source data where available. The full collection of summary dashboard plots is available in PalaeoWISE^26^. The overall completeness and accuracy of individual datasets was also verified during the creation of the LiPD files for each dataset.

### Relationship between proxies and hydroclimate

A key goal was to examine the extent to which the database captures the variability in hydroclimate using the state of Queensland as an example. However, a common challenge is that of stationarity, which assumes that the relationship between the proxy and climate variable over the shared period is representative of the entire time span of the proxy record. While methods exist to model unstable/nonlinear or multivariate relationships between proxies and climate variables, the approach adopted here is simple in the hope that it can be employed by a greater range of potential users, including the water industry, to efficiently screen the database for proxy data of relevance to catchment-scale hydroclimatic variability.

#### Selection of example proxy and hydroclimate variables

From the complete database, an example proxy set was selected for each of the eight archive types (sediment, foraminifera, ice core, leaf material, tree ring, ostracod, speleothem and coral) based on the highest correlation coefficient between the proxy, the 75 climate variables and 73 Queensland catchments. None of the ostracod-derived proxies reported a significant correlation coefficient with any of the selected climate variables and catchment, so no example is provided here. The data sets for the example proxy records are either continuous or have gaps/irregular time steps to allow us to test for changes in correlation coefficients based on record continuity, but all have an average temporal resolution of less than ten years.

A comprehensive set of hydroclimate variables relevant to catchment-scale hydroclimate modelling and future climate change projections (https://www.longpaddock.qld.gov.au/qld-future-climate/dashboard/) were selected: annual rainfall, evapotranspiration, temperature, Standardised Precipitation Index (SPI)^129,130^, Standardised Precipitation Evaporation Index (SPEI)^129^, and indices for severe and extreme wetness and dryness (Table 4). Gridded datasets (cell size = 0.05 degrees, approximately 10 km) of annual rainfall, evapotranspiration, and temperature were extracted from the Scientific Information for Landowners (SILO) database (https://www.longpaddock.qld.gov.au/silo) for the period 1889 to 2019 using the July to June water year. SPI and SPEI grids (cell size = 0.05 degrees) were then calculated from instrumental data at timescales of 12, 24, 36, and 48 months (Table 4), which are standard accumulation periods used by hydrologists and climatologists. In terms of hydrological applications annual and multi-annual time scales are important for water storages (and thus water supply security) because storages aggregate water over time and have variable ‘stress’ periods ranging from single to multiple years. These stress periods relate primarily to droughts, which in Australia are typically multi-year events. Periods of severe and extreme wetness and dryness were derived from all SPI and SPEI series using criteria outlined in Table 4 and are assessed over the same ~120-year period of recorded climate data. Catchment-averaged annual time-series for the 73 Queensland catchments were then derived from all climate grids for the July to June water year for the period 1/1/1889 to 31/12/2019.

**Table 4:** Overview of selected climate variables and their derivation periods.

Climatic Index	Description and use	Method	Reference	Derivation period
Average precipitation	Catchment-averaged precipitation (mm)	Annual precipitation averaged over each catchment.	^208^	12 months
Morton’s potential evapotranspiration	Catchment-averaged potential evapotranspiration	Morton’s equation, then averaged over each catchment.	^208^	12 months
Temperature	Catchment-averaged temperature (°C)	Annual temperature averaged over each catchment	^208^	12 months
Standardised Precipitation Index (SPI)	Identification of wetter and drier periods	Gamma distribution using a 1900–1999 reference period	^130,209^	12, 24, 36, and 48 months
Standardised Precipitation Evaporation Index (SPEI)	Identification of longer periods of aridity	Gamma distribution using a 1900–1999 reference period. Morton’s PET estimate.	^210–215^	12, 24, 36, and 48 months
SPI-flood index (Severe Floods)	Frequency of severe flooding	Number of consecutive months in a year with Standardised Precipitation Index ranging from 1.5 to 2.0	^130,216,217^	12, 24, 36, and 48 months
SPI-flood index (Extreme Flood)	Frequency of extreme flooding	Number of consecutive months in a year with Standardised Precipitation Index ≥2.0	^130,216,217^	12, 24, 36, and 48 months
SPI-drought index				
SPI-drought index (Severe Drought)	Frequency of severe droughts	Number of consecutive months in a year with Standardised Precipitation Index ranging from −1.5 to −2.0	^130,216,217^	12, 24, 36, and 48 months
(Extreme Drought)	Frequency of extreme droughts	Number of consecutive months in a year with Standardised Precipitation Index ≤−2	^130,216,217^	12, 24, 36, and 48 months

#### Outlier analysis of proxy data

As correlation calculations are not resistant to outliers in the proxy data, technical validation also tested for outliers using Rosner’s test^131^ in the R package EnvStats^132^. This procedure allows the user to test for multiple outliers in a dataset, as opposed to more static approaches using only a single outlier at a time. We note that the Rosner’s test does not take into account the temporal structure of the data, though there are other methods for finding outliers in such series (e.g. Chen and Liu (1993)). However, these are considerably more complex to implement in irregularly sampled series^133–136^.

**Fig. 3:**
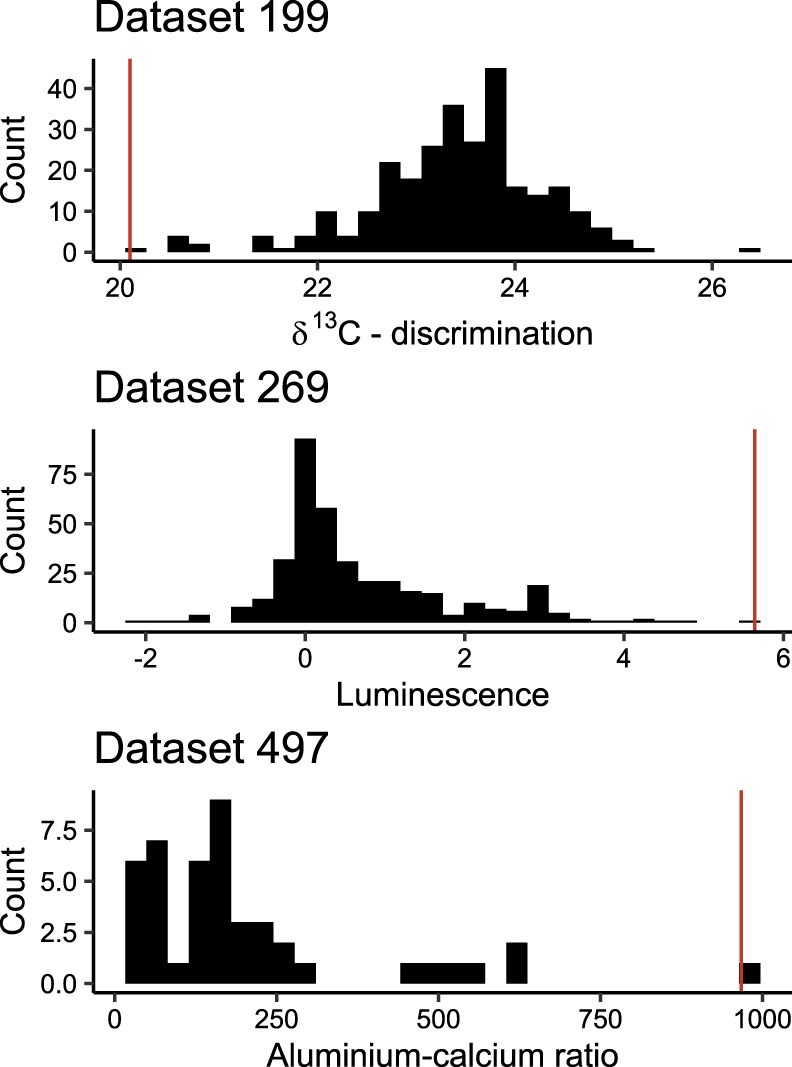
Selected plots for three proxy datasets that show the identified outliers in vertical red lines. Rosner’s test was applied to the entire proxy database, see the fieldnames file in PalaeoWISE^26^ for quality codes.

**Fig. 4:**
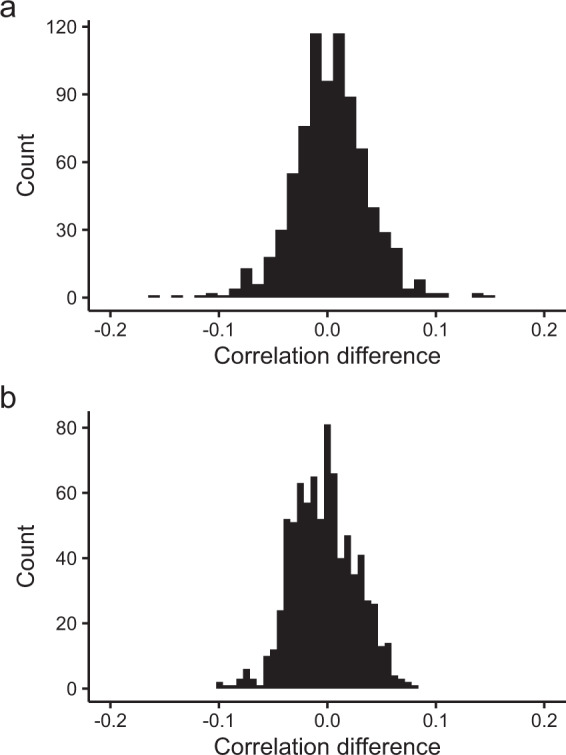
Outlier analysis of climate data. Histograms of the difference between the kernelised correlation coefficient when run on the raw data (Pearson) against the ranked data (Spearman) for catchment-averaged rainfall (**a**) and catchment-averaged temperature (**b**). Very few of the differences are observed outside the range (−0.1. 0.1).

A maximum of three outliers were tested on each of the example seven proxy datasets (Fig. 3) and two climate time series (annual rainfall and temperature; Fig. 4). Of the 2,156 proxy observations considered, the procedure found only three potential outliers, shown as vertical lines in Fig. 3. The identification of these outliers does not mean that they are incorrect, and remain included, but they might require some further investigation in any subsequent analysis. None of the data points extracted for the climatic observations were considered outliers. Beyond the seven records presented here as examples, the entire proxy database was quality controlled, with outliers identified using the method described above. The quality codes for outliers, suspected outliers, and missing values are detailed in PalaeoWISE (in both the LiPD metadata files and the fieldnames spreadsheet)^26^.

#### Temporal correlations

The relationship between the proxy records and catchment-averaged hydroclimate time series was tested using correlation analysis across the whole database. Correlation coefficients were determined using a kernel-based approach which is similar to Pearson’s correlation coefficient but has the advantage of applying to irregularly spaced data. The approach was used previously in Roberts *et al*. (2017;2020). For unevenly spaced series, Pearson’s correlation is not appropriate and the correlation method (and Python/Fortran code) from Rehfeld and Kurths (2014) was used. Conservative correlation lags of −5 to +5 years are included to acknowledge the potential for some dating uncertainty in high resolution proxies.

An approximate test for significant correlation is given as $> \frac{{z}_{\alpha /2}}{\sqrt{{N}^{* }}}$, where *z* is the inverse Gaussian distribution, α is the significance level and N* is the minimum number of data points for either time series within the overlapping period. Exact significance tests are not known for the Gaussian kernel method and the number of overlapping points changes depending on the lag and irregularity of the spacing of the two datasets being correlated^137^. Additionally, the significance tests also depend on the characteristics of the data series, for example those that are nonlinear, heteroskedastic or have a hidden dependence structure. This approximate significance test was applied to all correlation results presented here, and non-significant correlations are not presented.

To test the robustness of the Roberts *et al*. (2017) kernelised approach, we re-calculated the correlation coefficients based on the ranks for the data values. This in effect allows for a comparison of Pearson vs Spearman-type correlation where highly non-linear relationships would appear as a large difference between them. The differences between the Spearman and Pearson-type correlations when run on the same data sets showed very few values outside the range (−0.1, 0.1) (Fig. 4). The supplementary material within PalaeoWISE (Supplementary material; Section 2)^26^ includes a comparison of the Roberts *et al*. (2017;2020) approaches, the Rehfeld and Kurths (2014) approach, and Spearman and Pearson’s equations.

#### Visualising temporal correlations

**Fig. 5:**
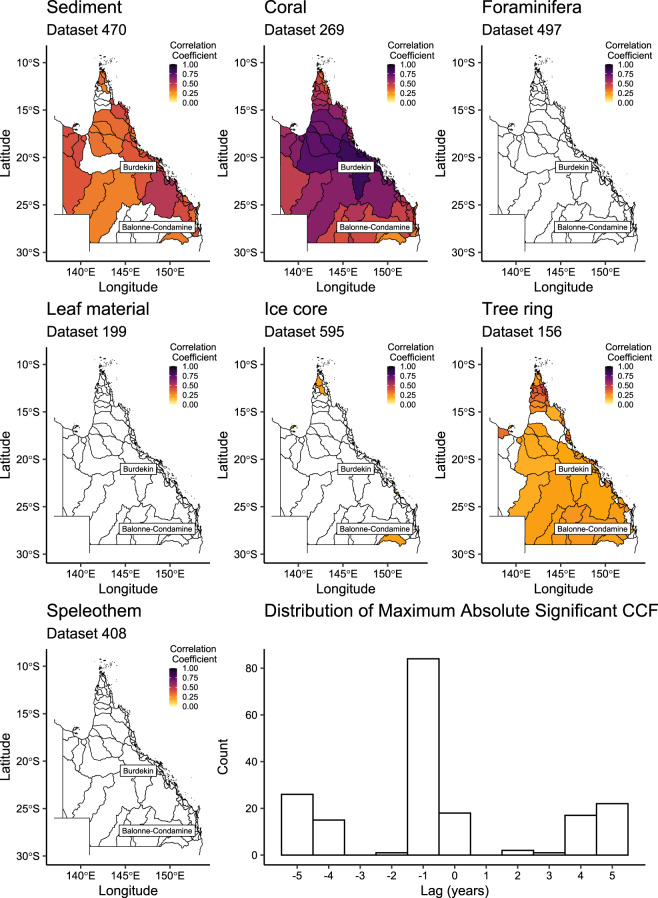
Correlation coefficients (ccf) shown are the maximum absolute ccf between catchment-averaged rainfall and the example proxies for all Queensland catchments from lags +5 to −5 years. White = non-statistically significant. Histogram shows the distribution of maximum absolute ccf by lag. The Burdekin and the Balonne-Condamine catchments referred to in the text are illustrated. Vector map data sourced from www.qldspatial.information.qld.gov.au.

**Fig. 6:**
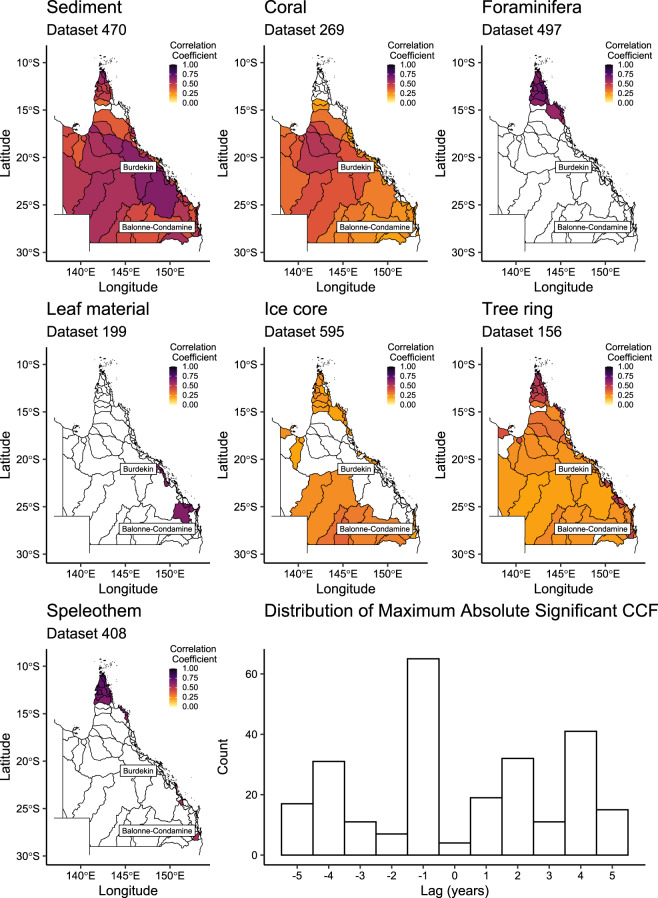
Correlation coefficients (ccf) between catchment-averaged temperature and the example proxies for all Queensland catchments from lags +5 to −5 years. White = non-statistically significant. Histogram shows the distribution of maximum absolute ccf by lag. Locations of the Burdekin and the Balonne-Condamine catchments referred to in the text are illustrated. Vector map data sourced from www.qldspatial.information.qld.gov.au.

Heat maps were constructed from the resultant correlation data to provide a condensed, visual tool that highlights the potential of individual proxies to reflect catchment-scale hydroclimate and the associated time lag (Figs. 5, 6). The heat maps display the maximum absolute correlation coefficients by climate index and catchment, with examples for catchment-averaged rainfall (Fig. 5) and temperature (Fig. 6) provided. Maps for each of the 75 hydroclimatic variables are available in a single page format, as are the correlation results for each catchment, dataset, and climate variable^26^. An interactive summary of the correlation results is also presented on the project website at www.palaeoclimate.com.au.

**Fig. 7:**
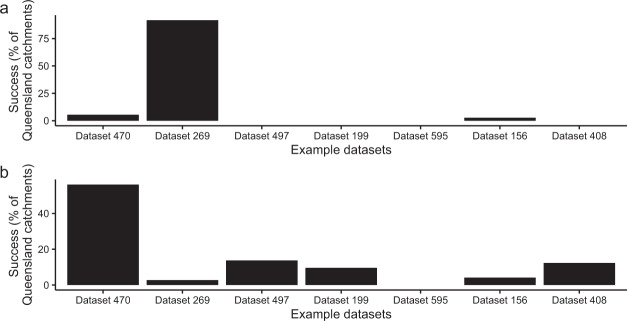
Identification of the most successful datasets for (**a**) catchment-averaged rainfall and (**b**) temperature. Success here is the proportion of the 73 Queensland catchments for which each proxy in the seven example datasets recorded the highest correlation coefficient at the 0.05% significance level. Similar plots for each climate variable are available in PalaeoWISE^26^.

The heat maps deliver meaningful information on the selection of proxy records and their associated skill with selected hydroclimate variables. This is especially valuable to appreciate the extent to which a given proxy correlates at the catchment (e.g., dataset 274), region (e.g., dataset 170; coastal eastern Queensland) or broader state-level (dataset 269) (Fig. 5). However, as heat maps are designed to show the ‘best case’ correlation coefficient, the lag is not constant across catchments. For example, a high correlation between catchment-averaged rainfall and proxy dataset 269 occurs at a lag of −1 in the Burdekin catchment (Fig. 5) but at a lag of +1 year in the Balonne-Condamine catchment (Fig. 5; PalaeoWISE correlations^26^). Despite the variability in associated lag, the majority of maximum absolute correlation coefficient values occur at lag −1 (Figs. 5, 6). To supplement the maps, and as an additional tool to aid the selection of relevant records, Fig. 7 shows the most ‘successful’ datasets for catchment-averaged rainfall and temperature records. Here, success was defined as the datasets with the highest significant absolute correlation coefficient for each of the 73 Queensland catchments for the climate variable of interest. Figure 7 shows dataset 269 has the largest number of highest correlations for rainfall, but that dataset 470 has the highest correlation coefficient for temperature within the Queensland catchments. Similar plots for each climate variable are presented in PalaeoWISE (success histograms)^26^.

## Usage Notes

Table 3 details the individual files contained within PalaeoWISE^26^. The current and all future versions of PalaeoWISE^26^ can be accessed at 10.6084/m9.figshare.14593863.v3, and the project website (www.palaeoclimate.com.au/project-outputs/proxy-map/access-the-palaeowise-database/). The proxy data contained in PalaeoWISE^26^ can also be accessed on NOAA WDS Paleoclimatology (https://www.ncdc.noaa.gov/paleo/study/34073)^32^ in both the LiPD format and also in WDS template text format for records not previously archived in this repository.

The approach and outputs are likely to be primarily used by the scientific community in the first instance to access both high- and low-resolution palaeoclimate proxy data in a single digital database. The inclusion of low- and high-resolution proxies facilitates use for hydrological modelling scenarios that may vary in timescales from annual or centennial.

PalaeoWISE^26^ also provides an essential resource for scientists and water managers to screen proxies correlated to hydroclimatic indices of their interest. The correlation approach is intended as an efficient, visual tool to identify relevant proxies and catchments for further investigation. The code accompanying this work allows for straightforward extrapolation of the approach to areas outside of Queensland where accompanying hydroclimate variables exist.

We welcome any additional or clarifying information to be incorporated into future versions. When using this database or any correlations presented within, please cite both the original data author(s)/collector(s) as well as this publication.

## Data Availability

Code to reformat the relational database to the LiPD and Rdata formats was adapted from this example (https://github.com/nickmckay/sisal2lipd) and is available in PalaeoWISE^26^. Code to produce the figures are available in PalaeoWISE^26^. Correlations were all produced using code published within the original publications cited within.
